# Two Cases of Fibroid Tumor of the Uterus Successfully Treated by Weak Currents of Galvanism

**Published:** 1891-10

**Authors:** J. A. Lyons

**Affiliations:** Chicago, Ill.


### TWO CASES OF FIBROID TUMOR OF THE UTERUS SUCCESSFULLY TREATED BY WEAK CURRENTS OF GALVANISM.*

BY J A. LYONS, M. D.

The following cases, which I have observed while assisting Dr. T. J. Watkins at his clinic in the Post-Graduate Medical

* Read before the Gynecological Society of Chicago, June, 1891.

School of this city, may be of interest, as they suffered from profuse and almost continuous metrorrhagia, and as their treatment consisted only in the use of weak currents of galvanism.

CASE I.—Mrs. C., 22 years of age, married four and one-half years; the mother of one child 3½ years old. She miscarried six months ago, at about the fourth month of gestation, at which time she suffered from puerperal septicemia and entered St. Luke's Hospital for treatment. During this time she flowed almost incessantly, and frequently hemorrhage was profuse. She continued to lose blood in this manner until January 30th of this year, when she applied for treatment at the dispensary of the Post-Graduate Medical School. Her health previous to the miscarriage was good, although her physique is slight and her temperament nervous. She came to the clinic for relief of metrorrhagia and of pain in the left inguinal region. She was excessively anemic and greatly debilitated.

Physical examination revealed a symmetrical enlargement of the uterus about the size of four months' gestation, which was diagnosed as an interstitial fibroid tumor. The depth of the uterus could not be ascertained, as a probe could not be passed beyond the os internum.

Galvanism was given as follows: 1. Apostoli's clay electrode was placed on the abdomen over the region of the tumor, and to this was fastened the negative pole of the battery. 2. The Apostoli intra-uterine electrode was introduced, but could only be passed to the internal os, and to this was attached the other pole of the battery. The electricity was now gradually administered until forty milliampères were given, when the patient experienced some pain. This current was continued for three minutes.

February 3d—that is, four days later—the above treatmen was repeated.

February 6th. Patient has had no hemorrhage since last treatment until this morning. The positive pole is again introduced into the uterus and the current increased to fifty milliampères.

February 10th. No hemorrhage since last treatment. The electrode is easily passed for the first time into the cavity of the uterus for four and one-half inches, and is made the negative pole of the battery. This treatment was repeated on February 13th, 17th and 20th.

February 27th. She has just completed a normal menstrual period. Fifty-five milliampères were given. The tumor is reduced to about three-fourths its original size. Her general condition is much improved.

March 20th. The galvanism has been given twice each week, as above described, and now the uterus is normal in size and position; the patient feels perfectly well and is discharged cured. Although the current given was weak and continued for only three minutes, yet at times she suffered so severely after the administration of the galvanism that she was forced to remain quiet in the hospital for two or three hours before returning to her home. About one month later, at our request, she returned to the dispensary feeling perfectly well, and examination again showed the uterus to be normal in size and position. In this case it is interesting to note that the metrorrhagia was cured while the electrode did not enter the uterine cavity.

Case II.—Mrs. C. Piper, living on North Robey street, American, aged 36 years and married eighteen years, has one child 17 years of age. She has had two miscarriages fifteen and thirteen years ago. For two years she has suffered from bearing-down pelvic pains and with severe pains in both inguinal regions and in back. Menstruation has been profuse for nearly two years, and has gradually become more painful, until now it is very severe, and for four months she has flowed almost continuously. The blood at times has been bright red and at other times dark and clotted.

Physical examination revealed a fibroid tumor of the uterus about the size of six months' pregnancy, which protrudes to the right and posterior. Galvanism was given practically as in Case I. The patient attended the clinic faithfully twice each week, although the treatment gave her but little if any relief for one month. Then the electrode was passed with difficulty into the cavity of the uterus. This could only be accomplished by placing the patient in the left lateral position, by exposing the cervix by means of a Sims' speculum, and straightening the uterus by traction on the cervix with a tenaculum. The canal was found to be very tortuous and six and one-half inches in depth. After this the resistance to the electrical current became much diminished, her hemorrhages abated, and her general condition rapidly improved. The strength of the current varied from thirty to seventy milliampères.

May 19—that is, forty-five days after the treatment was commenced, and fifteen days after the electrode was passed into the cavity of the uterus—the tumor is reduced about one-sixth in size and the patient is much improved.

June 9th. Patient is quite active and is now doing much of her own housework, while formerly she could attend to none of these household duties.

June 19th. Tumor is now about three-fourths its original size. Patient menstruates regularly, but not excessively and without suffering much pain. Her strength is practically restored and she now suffers but little from pelvic pain.

